# Sex-related differences in the effects of nutritional status and body composition on functional disability in the elderly

**DOI:** 10.1371/journal.pone.0246276

**Published:** 2021-02-02

**Authors:** Mika Sawada, Naoto Kubota, Rie Sekine, Mitsutaka Yakabe, Taro Kojima, Yumi Umeda-Kameyama, Satoshi Usami, Masahiro Akishita, Sumito Ogawa

**Affiliations:** 1 Department of Clinical Nutrition Therapy, The University of Tokyo, Tokyo, Japan; 2 Department of Diabetes and Metabolic Diseases, Graduate School of Medicine, The University of Tokyo, Tokyo, Japan; 3 Department of Geriatric Medicine, Graduate School of Medicine, The University of Tokyo, Tokyo, Japan; 4 Graduate School of Education, The University of Tokyo, Tokyo, Japan; University of New South Wales, AUSTRALIA

## Abstract

**Background:**

The aim of our study was to evaluate the influence of changes of nutritional status and body composition on the results of comprehensive geriatric assessment (CGA) in inpatients of a geriatric ward. Sex differences in these relationships were also investigated.

**Methods:**

A total of 212 elderly patients (>65 years old) admitted to the geriatric ward at the University of Tokyo hospital between 2012 and 2019 were enrolled in this study. CGA (ADL, IADL, MMSE, GDS, Vitality Index) was performed, along with assessment of body compositions (appendicular muscle mass, abdominal muscle mass, body fat mass) and blood malnutrition biomarkers (serum albumin, pre-albumin, 25-hydroxy vitamin D, zinc, hemoglobin concentrations).

**Results:**

Multiple linear regression analysis showed that upper, lower limbs and abdominal muscle masses were significantly associated with the score on ADL in men. On the other hand, abdominal muscle mass was negatively associated with the scores on GDS. Body fat mass was also negatively associated with the score on IADL. In contrast, in women, multiple linear regression analysis failed to show any significant associations between body composition parameters and scores on any domains of CGA. Unlike in men, however, blood malnutrition biomarkers were significantly associated with ADL, IADL, MMSE, and Vitality Index in women.

**Conclusions:**

Our study findings revealed that the association of the nutritional status and body composition with the functional status in the elderly differs by sex. These results suggest that intensification of exercise in men and improvement of the nutritional status in women are particularly useful to maintain the functional status.

## Introduction

With the aging of the world population, over the next three decades, the global population of older persons is projected to more than double, exceeding 1.5 billion, by 2050 [1]. In particular, aging of population is occurring most rapidly in Asia, where more than half of the elderly in the world are concentrated, representing an important issue that should be addressed urgently.

The elderly tend to exhibit multimorbidity and functional decline, which are believed to be correlated with each other [2–4]. It is important not only to provide appropriate treatment for the multimorbidity but also to perform a comprehensive functional assessment of the elderly. These functions can be evaluated with comprehensive geriatric assessment (CGA), which is defined as a multidimensional interdisciplinary diagnostic process focused on medical, psychological and functional capabilities of frail elderly in order to develop a coordinated plan for treatment and long-term care [5]. In practice, CGA involves evaluation of physical, cognitive and environmental functions including activities of daily living (ADL), instrumental activities of daily living (IADL), cognitive impairment, depression, and vitality, respectively [6], leading to improvement of the survival and functioning of the elderly [7–9].

Malnutrition is one of the critical factors that could be related to poor outcomes among the elderly population. Evaluation of elderly malnutrition includes assessment of blood biomarkers and body composition [10]. Albumin, pre-albumin, and hemoglobin have been widely used as representative markers of nutritional status, and lower serum albumin, pre-albumin, and hemoglobin levels are also known to be associated with malnutrition and impairment of ADL. Recently, both vitamin D and zinc levels have been highlighted as additional nutritional makers related to cognitive impairment and functional decline [11–13].

In addition, body composition alters with age, represented by a decrease in lean body mass together with an increase in body fat [14]. Previous studies have suggested that while an age-related increase in the body fat area was observed in both men and women [15], the decrease in skeletal muscle mass with advancing age was significantly more pronounced in men than in women [16,17]. These age-related changes of body composition were also reported to be associated with higher risk of hip fracture and mortality [18–20], further suggesting that age-related alterations of malnutrition biomarkers and body composition might be associated with functional disability and poorer prognosis. Nevertheless, it appears that sex-differences of the nutritional status, including biomarker levels and body composition data, on the domains of CGA have not been focused so much thus far.

Taken together, the aim of our study was to evaluate the association of the nutritional status, as assessed from biomarker levels and body composition data, with domains of the CGA, including physical and cognitive function, in elderly inpatients.

## Materials and methods

### Participants

This study was carried out in 212 elderly patients (>65 years old) admitted to a geriatric ward at the University of Tokyo hospital between 2012 and 2019. Major aim of the admission was to assess cognitive function, examine dizziness and gait disturbance, and/or control glycemia. We excluded patients who were admitted to hospital because of heart failure, edema or dehydration, since body composition as assessed by bioelectrical impedance analysis (BIA) system is affected by the hydration status. We also excluded patients who needed treatment in specialized units and/or with serum C-reactive protein (CRP) levels of >3 mg/l, because these may also be associated with significant alterations of nutritional status. Written informed consent was obtained from all the participants and/or their immediate family members, and the study was conducted with the approval of the Research Ethics Committee of the University of Tokyo Hospital (3811, 11707), in accordance with the principles of the Declaration of Helsinki; every effort was made to ensure patient anonymity.

### CGA assessment

CGA included the following five evaluations. Functional status in terms of ADL was assessed by Barthel index [21], where a score of 100 indicated the patient was independent with no need for assistance from others [22]. IADL was assessed by Lawton & Brody scale [22], where IADL score ranged from 0–5 points in men and 0–8 points in women [23]. Cognition was assessed by Mini Mental State Examination (MMSE), with scores of ≦23 denoting mild cognitive impairment [24,25]. Mood was assessed by geriatric depression scale-15 (GDS-15), with scores of ≧6 denoting depression [26,27]. ADL-related vitality was assessed by Vitality Index, with scores of ≦7 implying lower survival rates [28]. CGA assessment was performed by senior geriatricians.

### Body composition

We used the BIA system (InBody S10, InBody Japan) to evaluate the appendicular muscle mass, abdominal muscle mass and body fat mass of the participants. The system separately measured the impedance in the subjects’ right arm, left arm, trunk, right leg, and left leg at six different frequencies (1, 5, 50, 250, 500 and 1000 kHz) for each body segment. Body composition analysis was performed at ambient temperature in accordance with the recommended BIA measurement conditions, as follows; (1) fasting for 4 hours and no alcohol for 8 hours prior to the measurements; (2) bladder voided prior to the measurements; (3) no exercise for 8 hours prior to the measurements [29]. BIA assessment was carried out between 4 pm and 6 pm. We use the absolute values of muscle and fat mass, since recent studies demonstrated that the absolute values of muscle and fat mass were associated with risk factors for cognitive impairment and cardiovascular disease or all-cause mortality in elderly people [30–32].

### Blood biomarkers

Blood biochemical parameters of nutrition were analyzed, including serum albumin, pre-albumin, 25-hydroxy vitamin D (25(OH)D), zinc, and hemoglobin concentrations. Blood samples were collected from all participants at admission.

### Statistical analysis

Results are expressed as means ± standard deviation (SD). Comparisons between groups were performed by Student’s *t*-test for two samples. Correlations between body composition parameters and scores on the CGA domains or between blood biomarkers and scores on the CGA domains were analyzed by calculation of Pearson correlation coefficients. Multiple linear regression analysis using CGA as the dependent variable was performed to identify the determinants of scores on the CGA domains among potential factors. Independent variables with a variance inflation factor of less than 2 were adopted to avoid the multicollinearity problem [33,34]. We also confirmed normality of residuals for each dependent variable through histograms [33,34]. *P* values of less than 0.05 were considered as being indicative of statistical significance. Data were analyzed using SPSS version 22 (IBM SPSS Statistic Version 22).

## Results

### Characteristics of the study participants according to sex

A total of 212 subjects were included in the study, including 98 men (46.2%) and 114 women (53.8%); average age of men was 80.4 ± 9.3 years, while that of women was 81.8 ± 6.4 years (Table 1). Average body mass index (BMI) was 22.1 ± 3.7 kg/m^2^ in men and 21.9 ± 3.7 kg/m^2^ in women. Consistent with previous reports [15,17], appendicular muscle mass was higher in men, while body fat percentage was higher in women.

**Table 1:** Characteristics of the study participants according to sex.

Variable	All (n = 212)	Men (n = 98)	Women (n = 114)	*P*-value
Age (y)	81.2 ± 7.9	80.4 ± 9.3	81.8 ± 6.4	0.174
Body composition				
Body mass index (kg/m^2^)	22.0 ± 3.7	22.1 ± 3.7	21.9 ± 3.7	0.632
Upper limb muscle mass (kg)	3.24 ± 1.06	4.06 ± 0.84	2.58 ± 0.69	
Lower limb muscle mass (kg)	12.4 ± 4.1	15.3 ± 3.3	9.8 ± 2.8	
Abdominal muscle mass (kg)	15.4 ± 3.5	18.2 ± 2.5	13.1 ± 2.2	
Body fat mass (kg)	14.7 ± 6.4	14.7 ± 6.3	14.8 ± 6.6	0.991
Body fat percentage (%)	27.4 ± 9.2	24.2 ± 7.8	30.1 ± 9.4	<0.01
Blood biomarkers				
Albumin (g/dl)	3.8 ± 0.4	3.8 ± 0.4	3.7 ± 0.4	0.750
Pre-Albumin (mg/dl)	21.7 ± 5.7	22.2 ± 6.2	21.2 ± 5.1	0.223
25-hydroxy vitamin D (nmol/L)	55.1 ± 17.9	57.6 ± 18.0	53.3 ± 17.8	0.173
Zinc (μg/dL)	66.8 ± 16.5	70.3 ± 15.2	64.5 ± 17.1	0.121
Hemoglobin (g/dL)	12.2 ± 1.7	12.7 ± 1.9	11.8 ± 1.4	
CGA				
ADL (score)	87.4 ± 17.8	88.4 ± 18.0	86.6 ± 17.7	0.485
IADL (score)		3.8 ± 1.5	5.4 ± 2.3	
MMSE (score)	22.9 ± 5.2	23.6 ± 4.8	22.3 ± 5.5	0.071
GDS (score)	5.9 ± 3.8	5.4 ± 3.6	6.3 ± 4.0	0.079
Vitality Index (score)	8.8 ± 1.5	8.9 ± 1.5	8.8 ± 1.5	0.818

Data are expressed as the means ± SD. CGA, Comprehensive Geriatric Assessment; ADL, activities of daily living; IADL, instrumental activities of daily living; MMSE, Mini Mental State Examination; GDS, geriatric depression scale-15. p -values were calculated by Student’s t-test for two-samples.

Serum concentration of 25(OH) D was 57.6 ± 18.0 nmol/L in men and 53.3 ± 17.8 nmol/L in women on average, respectively. Mean serum zinc concentration was 70.3 ± 15.2 μg/dL in men and 64.5 ± 17.1 μg/dL in women, both of which, less than 80 μg/dL, were regarded as malnutrition-related zinc deficiency [35].

The overall mean ADL score was 87.4 ± 17.8, and almost half of the participants (n = 116) had functional limitations (ADL scores < 95). The mean IADL score was 3.8 ± 1.5 in men and 5.4 ± 2.3 in women, respectively, indicating that most of the participants (men: n = 48, women: n = 65) needed some assistance (IADL scores <5 in men and <8 in women). The mean MMSE score was 22.9 ± 5.2; almost half of the subjects (n = 100) had mild cognitive impairment (MMSE scores < 24). The mean score on GDS was 5.9 ± 3.8, and almost half of the participants (n = 101) had depression (GDS scores ≥ 6). The mean Vitality Index was 8.8 ± 1.5, and almost 20% of the patients (n = 38) showed impaired ADL-related vitality (Vitality Index ≤ 7). There was no significant difference in the average score on the GDS or the Vitality Index between the men and women included in the study.

### Associations between body composition parameters and scores on the CGA domains in men and women

In male patients, upper, lower limb and abdominal muscle masses were significantly positively associated with scores on ADL (*P*<0.01), IADL (*P*<0.05), and Vitality index (*P*<0.01), whereas they were negatively associated with the score on GDS (*P*<0.05) (S1 Table). Lower limb muscle mass was positively associated with MMSE score (*P*<0.01) (S1 Table). These results suggest the associations of various body composition parameters with scores on the CGA domains in men. Multiple linear regression analysis in men demonstrated that upper, lower limb and abdominal muscle masses were significantly positively associated with the score on ADL (Table 2). On the other hand, body fat mass was negatively associated with the score on IADL. Abdominal muscle mass was also negatively associated with the score on GDS, suggesting that more abdominal muscle mass is associated with better psychological condition. In contrast, in women, correlation analysis revealed association of lower limb mass with the score on IADL (*P*<0.01) (S1 Table), whereas multiple linear regression analysis failed to reveal any significant positive associations between the variables and any scores on the CGA domains (Table 2).

**Table 2:** Multiple linear regression analysis to identify unique associations between body composition parameters and scores on the CGA domains.

Variable	ADL†		IADL‡		MMSE§		GDS||		Vitality Index¶	
	β	*P*-value	β	*P*-value	β	*P*-value	β	*P*-value	β	*P*-value
Men										
Upper limb muscle mass	0.430**	0.005	-0.193	0.166	-1.24	0.294	-0.165	0.109	0.227	0.076
Lower limb muscle mass	0.291*	0.034	0.233	0.071	0.129	0.242	-0.047	0.672	0.031	0.787
Abdominal muscle mass	0.468**	0.002	-0.197	0.154	-0.076	0.520	-0.219*	0.034	0.134	0.291
Body fat mass	0.010	0.948	-0.324*	0.035	-0.202	0.877	-0.202	0.859	0.250	0.069
Women										
Upper limb muscle mass	-0.021	0.875	0.035	0.828	-0.001	0.993	0.005	0.965	0.125	0.342
Lower limb muscle mass	-0.105	0.442	0.310	0.056	0.057	0.634	-0.027	0.779	-0.066	0.610
Abdominal muscle mass	-0.055	0.687	0.103	0.516	0.013	0.913	0.008	0.941	0.115	0.378
Body fat mass	-0.138	0.307	-0.043	0.787	0.164	0.171	0.000	0.997	-0.006	0.966

Multiple linear regression analysis was performed using CGA as the dependent variable, to identify the determinants of the scores on the CGA domains among potential factors.

*Significant at P <0.05,

**Significant at P <0.01.

^β^ denotes standardized regression coefficient.

ADL, activities of daily living; IADL, instrumental activities of daily living; MMSE, Mini Mental State Examination; GDS, geriatric depression scale-15.

### Associations between blood malnutrition biomarkers and scores on the CGA domains in men and women

In male subjects, higher serum albumin and 25(OH) D levels were significantly associated with higher scores on ADL respectively (*P*<0.05) (S2 Table). Serum pre-albumin level was also positively associated with the score on IADL (*P*<0.05) (S2 Table). However, multiple linear regression analysis failed to reveal any such associations (Table 3). In women, serum levels of albumin, pre-albumin, zinc, and hemoglobin showed positive associations with scores on ADL (P<0.01), IADL (P<0.01), and Vitality Index (P<0.05) (S2 Table). Serum levels of albumin and hemoglobin also significantly associated with MMSE score (P<0.05) (S2 Table). Multiple linear regression analysis revealed a significant association between serum levels of albumin and the score on ADL (Table 3). Serum levels of albumin and hemoglobin remained significant determinants of the score on IADL, while those of pre-albumin and zinc revealed no significant associations with the score on IADL. Significant association was also seen between serum hemoglobin levels and MMSE score. Higher serum albumin and pre-albumin levels were also associated with higher scores on Vitality Index. These results suggest that the associations between blood malnutrition biomarkers and scores on the CGA domains were stronger in women than in men.

**Table 3:** Multiple linear regression analysis to identify unique associations between blood biomarkers and scores on the CGA domains.

Variable	ADL†		IADL‡		MMSE§		GDS||		Vitality Index¶	
	Β	*P*-value	β	*P*-value	β	*P*-value	β	*P*-value	β	*P*-value
Men										
Albumin	0.286	0.071	-0.202	0.892	0.129	0.307	0.056	0.611	-0.230	0.088
Pre-albumin	0.167	0.300	0.149	0.330	-0.071	0.584	0.012	0.911	-0.081	0.553
25(OH)D	0.407	0.084	-0.265	0.174	0.288	0.079	-0.274	0.061	-0.153	0.447
Zinc	0.130	0.668	-0.074	0.787	0.371	0.134	-0.215	0.389	-0.441	0.130
Hb	0.038	0.817	-0.070	0.649	0.114	0.383	0.071	0.529	0.122	0.380
Women										
Albumin	0.267*	0.014	0.297*	0.021	-0.124	0.212	-0.009	0.917	0.216*	0.046
Pre-albumin	0.005	0.976	0.129	0.399	-0.124	0.287	-0.018	0.863	0.274*	0.048
25(OH)D	0.101	0.516	0.177	0.373	-0.073	0.617	-0.042	0.741	-0.055	0.737
Zinc	0.390	0.117	0.127	0.615	0.026	0.884	0.110	0.510	-0.044	0.837
Hb	-0.034	0.771	0.365*	0.010	0.236*	0.027	0.022	0.815	-0.031	0.794

Multiple linear regression analysis was performed using the score on the CGA as the dependent variable to identify the determinants of the CGA scores among potential factors.

*Significant at P <0.05.

^β^ denotes standardized regression coefficient.

25(OH)D, 25-hydroxy vitamin D; Hb, hemoglobin; ADL, activities of daily living; IADL, instrumental activities of daily living; MMSE, Mini Mental State Examination; GDS, geriatric depression scale-15.

## Discussion

The objective of this study was to examine whether body composition and blood malnutrition biomarkers were associated with scores on the CGA domains in elderly subjects. While our results suggested the existence of an association between the body composition and physical functional status in men, blood malnutrition biomarkers were associated with all of the physical functional status, cognitive function, and ADL-related vitality in women.

In the present study, significant associations were observed in men, but not in women, between upper, lower limb and abdominal muscle masses and physical functions such as ADL and GDS. While the muscle mass is well-known to be relatively higher in men, the rate of age-related loss of muscle mass is also known to be higher in men than in women. In a previous study examining skeletal muscle mass and its distribution, the rate of decrease in upper and lower limb muscle masses with age was significantly greater in men than in women [16]. Similar results that more rapid decrease in muscle masses in men were also reported in Japanese, where the absolute rates of change in both total and appendicular muscle masses were consistently larger in men compared with women during 20 to 79 years of age [36]. Thus, loss of muscle mass is likely to affect the physical functional status to a greater degree among elderly men compared with elderly women.

Upper limb, lower limb and abdominal muscles are known to play overlapping and distinct roles in physical activities. In this study, we also investigated the relationship between each of the three muscles and CGA domains, and found that all of them were associated with ADL in men, further indicating that abdominal muscle mass was significantly associated with depressive state (Table 2). These results suggest that keeping all these muscles might be important for the maintenance of both physical and psychological functions in elderly people. Further studies are expected to reveal underlying mechanism of the relationship between abdominal muscle mass and depression in elderly men.

Multiple linear regression analysis also revealed the existence of a significant association between blood hemoglobin level and scores on IADL and MMSE in women (Table 3). Consistent with this finding, a previous cohort study demonstrated an association between malnutrition-related anemia and impairment of IADL only in women, not in men [37]. It was also reported that malnutrition-related anemia is an independent risk factor for dementia [38] and that elderly women, but not men, with dementia had a higher prevalence of iron deficiency anemia [39]. Taken together, elderly women with low hemoglobin levels due to malnutrition are more likely to exhibit lower scores on MMSE and IADL.

It is also well known that prevalence of osteoporosis and dementia are higher in women compared with men, and that malnutrition are potential risk factors of both of the diseases [40,41]. In addition, decrease in ADL, IADL and MMSE scores are also known to be associated with risk of osteoporosis and dementia, respectively [40,42]. Taken together with our findings that malnutrition was associated with decrease in ADL, IADL and MMSE scores only in women, nutritional assessment and its improvement might play an important role in maintaining physical and cognitive function mostly in women. Verbrugge defined the contradiction of higher female morbidity but higher male mortality as "sex morbidity-mortality paradox" [43]. It is also suggested that sex differences found in this study where domains of the CGA were associated with body composition in men and nutritional status in women might contribute to the underlying background of the paradox.

There were some limitations of this study. First, due to its cross-sectional study design, we could not clarify the causal relationship between the changes in nutritional status/body composition and functional status of the subjects. Second, because of the relatively small sample size, our results may not well represent the general Japanese older adult population. Third, sensitivity of BIA used in the present study is not well validated and BIA system may not yield entirely accurate body composition parameters. In practice, abnormal hydration status or extremes of body weight might also influence the measurements [44]. Forth, due to a lack of information on sociodemographic factors such as education and marital status, multiple linear regression analysis was not adjusted for these variables. To clarify age-related changes of nutritional status and body composition together with its sex differences, further investigation using a larger sample size including populations of various ethnicities is expected.

In conclusion, our study suggest that there exist sex differences in the association of the nutritional status and body composition with the functional status in the elderly. While significant associations were observed between the body composition parameters and physical function in men, blood malnutrition biomarker levels were associated with all of the physical functional status, cognitive function, and ADL-related vitality in women. Physical exercise and appropriate nutrition are important measures in older people to reduce the incidence of comorbidities and prevent disability, further suggesting that intensification of exercise in men and improvement of the nutritional status in women are particularly useful to maintain the functional status.

## Supporting information

S1 Table Correlations between the body composition parameters and scores on the CGA domains. (DOCX)

S2 Table Correlations between blood biomarkers and scores on the CGA domains. (DOCX)
