# Supplementary material for: Sex-related differences in the effects of nutritional status and body composition on functional disability in the elderly
Source: PLoS One. 2021 Feb 2;16(2):e0246276. doi: 10.1371/journal.pone.0246276 (PMC7853464; doi:10.1371/journal.pone.0246276)
Supplement: S1 Table — (DOCX) [file pone.0246276.s001.docx]

**S1 Table**

**Correlations between the body composition parameters and scores on the CGA domains**

| Variable | ADL | IADL | MMSE | GDS | Vitality Index |
| --- | --- | --- | --- | --- | --- |
| Men | | | | | |
| Upper limb muscle mass | 0.415** | 0.268** | 0.082 | -0.253** | 0.390** |
| Lower limb muscle mass | 0.496** | 0.499** | 0.405** | -0.236* | 0.321** |
| Abdominal muscle mass | 0.431** | 0.264* | 0.107 | -0.267* | 0.352** |
| Body fat mass | 0.023 | -0.021 | -0.044 | -0.034 | 0.147 |
| Women | | | | | |
| Upper limb muscle mass | 0.046 | 0.084 | 0.025 | 0.001 | 0.096 |
| Lower limb muscle mass | 0.112 | 0.268** | 0.155 | -0.121 | 0.123 |
| Abdominal muscle mass | 0.061 | 0.130 | 0.068 | -0.018 | 0.125 |
| Body fat mass | -0.095 | -0.029 | 0.035 | 0.017 | -0.036 |

**Legends for figures**

**S1 Table**

**Correlation between the body composition parameters and scores on the CGA domains.**

Pearson correlation coefficients. *Significant at *P* <0.05, **Significant at *P* <0.01. ADL, activities of daily living; IADL, instrumental activities of daily living; MMSE, Mini Mental State Examination; GDS, geriatric depression scale-15.
