# Supplementary material for: Sex-related differences in the effects of nutritional status and body composition on functional disability in the elderly
Source: PLoS One. 2021 Feb 2;16(2):e0246276. doi: 10.1371/journal.pone.0246276 (PMC7853464; doi:10.1371/journal.pone.0246276)
Supplement: S2 Table — (DOCX) [file pone.0246276.s002.docx]

**S2 Table**

**Correlations between blood biomarkers and scores on the CGA domains**

| Variable | ADL | IADL | MMSE | GDS | Vitality Index |
| --- | --- | --- | --- | --- | --- |
| Men | | | | | |
| Albumin | 0.215* | 0.166 | 0.149 | 0.025 | -0.029 |
| Pre-albumin | 0.119 | 0.225* | 0.030 | -0.043 | 0.088 |
| 25(OH)D | 0.275* | 0.151 | 0.334 | -0.181 | 0.097 |
| Zinc | 0.117 | 0.121 | 0.277 | -0.085 | -0.094 |
| Hb | 0.133 | 0.115 | 0.145 | 0.023 | 0.144 |
| Women | | | | | |
| Albumin | 0.494** | 0.553** | 0.301* | -0.150 | 0.421** |
| Pre-albumin | 0.302** | 0.396** | 0.109 | -0.027 | 0.302** |
| 25(OH)D | 0.187 | 0.201 | 0.010 | -0.069 | 0.064 |
| Zinc | 0.459** | 0.396** | 0.189 | -0.007 | 0.324* |
| Hb | 0.306** | 0.473** | 0.411** | -0.059 | 0.287** |

**Legends for figures**

**S2 Table**

**Correlation between blood biomarkers and scores on the CGA domains.**

Pearson correlation coefficients. *Significant at *P* <0.05, **Significant at *P* <0.01. 25(OH)D, 25-hydroxy vitamin D; Hb, hemoglobin; ADL, activities of daily living; IADL, instrumental activities of daily living; MMSE, Mini Mental State Examination; GDS, geriatric depression scale-15.
